# Bicuspid pulmonary and bicuspid aortic valve in association with Gasul phenomenon (triple combination): a case report and literature review

**DOI:** 10.1186/s43044-025-00682-8

**Published:** 2025-09-08

**Authors:** Uma Devi Karuru, T. Naveen, Sai Kumar Mysore, Sadanand Reddy Tummala, Ashirbad Parhi, Kiran Kumar Kanjerla

**Affiliations:** 1 Nizam’s Institute of Medical Sciences, Hyderabad, India; 2 ESIC medical college and Super Speciality Hospital, Hyderabad, India

**Keywords:** Bicuspid aortic valve, Bicuspid pulmonary valve, Gasul phenomenon

## Abstract

**Background:**

Congenital heart disease (CHD) is a significant health concern affecting approximately 1% of live births. Among these anomalies, bicuspid aortic valve (BAV) is the most prevalent, while bicuspid pulmonary valve (BPV) remains exceptionally rare. This case report presents a unique instance of a 10-year-old girl diagnosed with the combination of BAV and BPV alongside a ventricular septal defect (VSD) and infundibular stenosis, referred to as the Gasul phenomenon.

**Case presentation:**

The patient, initially identified with a heart murmur during infancy, exhibited dyspnea classified as New York Heart Association (NYHA) class II but showed no cyanosis or other acute symptoms. Echocardiographic evaluation revealed a small restrictive VSD with significant left-to-right shunting, severe infundibular stenosis, and the coexistence of both BAV and BPV. Surgical intervention involved closing the VSD and resecting the hypertrophied infundibular muscle, leading to improved hemodynamics and symptomatic relief postoperatively.

**Conclusions:**

This case emphasizes the rarity and complexity of having both bicuspid valves in a single patient and the clinical challenges associated with the Gasul phenomenon. It highlights the importance of comprehensive echocardiographic assessment and timely surgical intervention in managing such congenital anomalies, ultimately improving long-term outcomes. The report contributes to the limited literature on simultaneous BAV and BPV diagnoses and underscores the need for heightened clinical awareness and diagnostic scrutiny in pediatric patients with congenital heart defects. Further studies are warranted to explore the natural history and management strategies for patients with this unusual combination of cardiac anomalies.

**Supplementary Information:**

The online version contains supplementary material available at 10.1186/s43044-025-00682-8.

## Background

Congenital heart disease (CHD) is one of the most prevalent birth defects globally, affecting approximately 1% of live births. CHD encompasses a wide range of structural defects that arise during embryonic development, often resulting in abnormal blood flow patterns within the heart and great vessels. Among Pediatric patients, ventricular septal defect (VSD) is one of the most frequently diagnosed congenital cardiac anomalies. However, in the general population, the bicuspid aortic valve (BAV) is the most common congenital heart defect, affecting around 1–2% of the population [1, 2]. In contrast, the bicuspid pulmonary valve (BPV) is an extremely rare condition, with few cases reported in the literature [3]. The rarity of BPV is such that many of these cases are only identified post-mortem or incidentally during surgical procedures.

Gasul phenomenon refers to a unique combination of congenital cardiac anomalies that includes the presence of a VSD, acquired right ventricular outflow obstruction (RVOTO), and other associated lesions, such as infundibular stenosis [4]. While Gasul phenomenon is rarely diagnosed, it is more frequently reported in developing countries, where limited access to early surgical interventions often delays diagnosis and treatment. This report presents a case of a pediatric patient diagnosed with Gasul phenomenon via two-dimensional echocardiography. The patient exhibited both bicuspid pulmonary and bicuspid aortic valves—an exceedingly rare combination.

## Case presentation

A 10-year-old girl was referred to our Pediatric cardiology clinic for further evaluation after a persisted heart murmur which initially detected in infancy. The murmur was first identified during a routine check-up at three months of age, and since then, the patient had been under medical management. Her primary symptom at presentation was dyspnea, classified as New York Heart Association (NYHA) class II. Notably, the patient had no history of cyanosis, heart failure, palpitations, syncope, or loss of consciousness.

The patient was the second child of non-consanguineous parents and was born full-term with a normal birth weight. She had no significant medical history apart from the diagnosed heart murmur and was performing well academically. No other family members had a history of congenital heart disease or significant cardiovascular abnormalities.

The patient’s growth and development were appropriate for her age. On physical examination, she was well-nourished and in no acute distress. Cardiovascular examination revealed a grade III/VI systolic murmur heard best over the left lower sternal border. There was no evidence of cyanosis, clubbing, or peripheral edema.

## Investigations

Initial laboratory tests, including complete blood count (CBC), liver function tests (LFTs), and renal function tests (RFTs), were all within normal limits. Viral markers for common pediatric infections, including cytomegalovirus (CMV) and rubella, were negative. Genetic testing was negative for syndromic association. Chest radiography revealed moderate cardiomegaly with a cardiothoracic ratio of 60%, predominantly due to right ventricular enlargement. Electrocardiography (ECG) showed normal sinus rhythm with signs of right ventricular hypertrophy (RVH), but there were no arrhythmias or conduction delays.

Echocardiographic evaluation was performed using two-dimensional echocardiography (2D ECHO), which revealed the presence of a small restrictive ventricular septal defect (VSD) with a significant left-to-right shunt (Video 01). The gradient across the VSD was 88 mm Hg, indicating a high pressure difference between the left and right ventricles. Additionally, severe infundibular stenosis was observed, with a peak gradient across the right ventricular outflow tract (RVOT) was 60 mm Hg (Video 02). The great arteries were normally related without any significant aortic or main pulmonary artery dilatation, ruling out more complex congenital anomalies such as transposition of the great arteries.

**Fig. 1:**
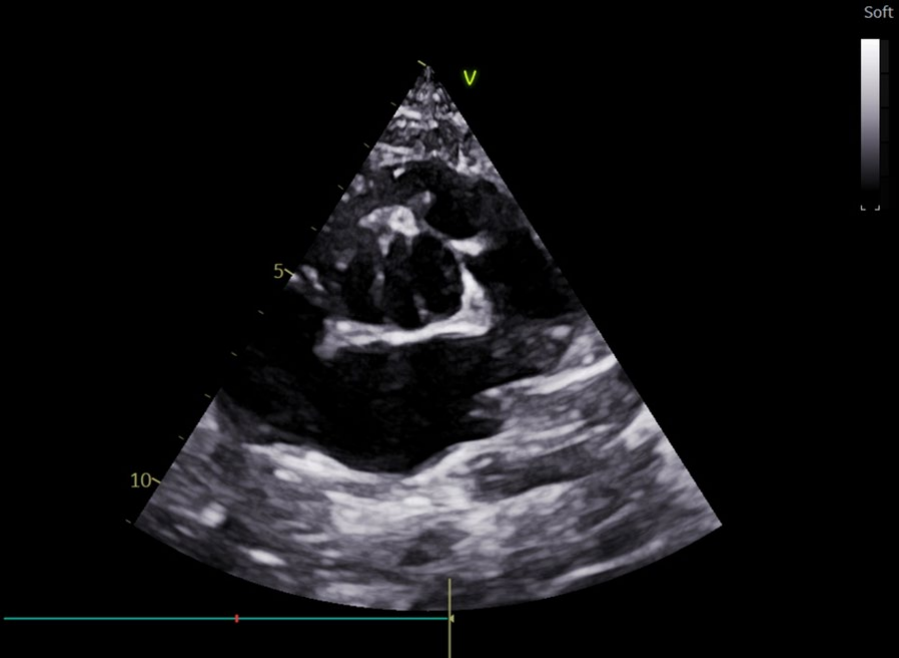
Parasternal short axis view at level of aortic valve showing bicuspid aortic valve without raphe in open position

**Fig. 2:**
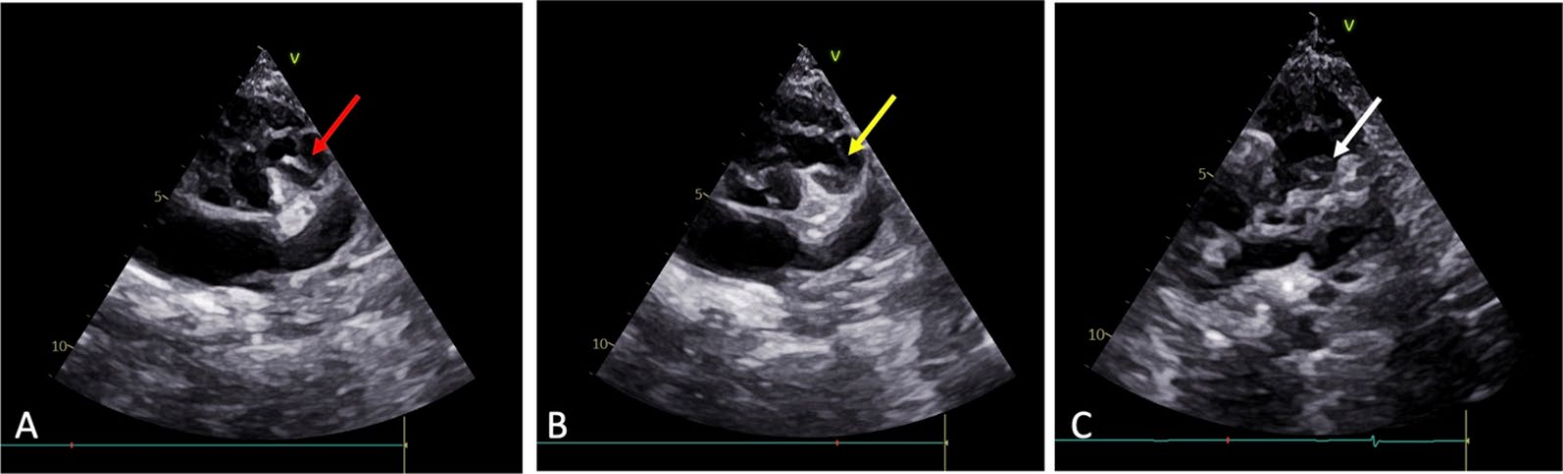
Modified Parasternal short axis view at level of aortic valve showing bicuspid pulmonary valve in Closed position (**A**, red arrow), doming valve in open position (**B**, yellow arrow) and horizontally oriented valve (**C**, white arrow)

Importantly, the echocardiogram also revealed the presence of both a bicuspid aortic valve (BAV) and a bicuspid pulmonary valve (BPV). The BAV was type B without evidence of a raphe (an incomplete fusion of the cusps) (Fig. 1), while the BPV showed evidence of pulmonary valve doming, a hallmark of valvular stenosis (Fig. 2). There was mild right ventricular hypertrophy (RVH), likely secondary to the combination of increased RV pressure from the VSD and the infundibular stenosis. Cardiac catheterization demonstrated a significant oxygen step up at the ventricular levels, RVOT gradient of 55 mm Hg and normal systemic arterial pressures and oxygen saturations.

Given the patient’s hemodynamic status and the progression of her symptoms, surgical intervention was deemed necessary. The patient underwent surgery to close the VSD with a pericardial patch and resect the hypertrophied infundibular muscle under cardiopulmonary bypass. Intraoperative findings confirmed the presence of both bicuspid aortic and pulmonary valves, as well as the VSD and infundibular stenosis. The patient had an uneventful postoperative course and was discharged from the hospital one week after surgery.

At her 1-month follow-up visit, the patient reported significant improvement in her exercise tolerance and was now classified as NYHA class I. Repeat echocardiography demonstrated a well-positioned VSD patch with minimal residual gradient across the right ventricular outflow tract. Both the bicuspid aortic and pulmonary valves continued to function normally, with no significant regurgitation and stenosis observed postoperatively.

## Discussion

The combination of bicuspid pulmonary and bicuspid aortic valves in a single patient is an exceptionally rare congenital anomaly. In most reported cases, bicuspid valves are associated with other complex congenital heart defects, such as Tetralogy of Fallot (TOF), double-outlet right ventricle (DORV), or transposition of the great arteries (TGA). This case is unusual in that the patient had both bicuspid aortic and pulmonary valves in the absence of these more complex anomalies, apart from the VSD and infundibular stenosis.

Bicuspid aortic valve (BAV) is relatively common, with a prevalence of 1–2% in the general population. It is often associated with progressive aortic dilation, aortic stenosis, or regurgitation over time. In contrast, bicuspid pulmonary valve (BPV) is much rarer, with only a handful of cases reported in the literature. BPV may also be associated with pulmonary stenosis or regurgitation, but the natural history of BPV is less well understood than that of BAV.

In the context of Gasul phenomenon, the combination of a VSD with an acquired muscular ridge across the RVOT presents a unique clinical challenge. Gasul and his colleagues first described this phenomenon in the 1950s when they noted that some patients with VSD developed RVOTO over time due to the formation of a hypertrophied muscle bundle across the outflow tract. This process is thought to result from crista supra ventricular is hypertrophy, distortion of the parietal and septal bands, and progressive infundibular stenosis. In this patient, early surgical intervention to close the VSD and resect the infundibular muscle likely prevented the development of more severe RVOTO, which could have led to cyanosis or heart failure.

The rarity of bilateral bicuspid valves (both aortic and pulmonary) being diagnosed during life is further emphasized by a review of post-mortem cases. In a study of 84 post-mortem cases of bicuspid aortic valves associated with congenital heart disease (CHD), 14 patients (17%) were found to have abnormalities of the pulmonary valve. Of these, 8 cases (10%) had bicuspid pulmonary valves, and 6 of these patients also had a perimembranous VSD [5]. These findings suggest that bicuspid pulmonary valves may be more common in association with other congenital heart defects, such as VSD, but that these anomalies are often underdiagnosed during life.

The literature on live pediatric cases of patients with both bicuspid aortic and pulmonary valves is exceedingly limited. In one case series, only four live pediatric patients with both BAV and BPV were reported [6]. Of these, only one case was associated with a small, mid-muscular VSD. This particular case also involved severe pulmonary valve stenosis, which required balloon dilatation of the pulmonary valve at 11 months of age. None of the cases described in the literature exhibited features consistent with Gasul phenomenon, further highlighting the uniqueness of this case.

Interestingly, there is a case report of a 47-year-old adult with both BAV and BPV who also had a large atrial septal defect (ASD) [7]. This patient underwent successful surgical closure of the ASD with a patch, further emphasizing the rarity of this combination of anomalies in both pediatric and adult populations. This case underscores the importance of considering the coexistence of multiple congenital anomalies, even in older patients, as these defects may remain undiagnosed for many years.

## Conclusion

This case report highlights the rare coexistence of bicuspid pulmonary and aortic valves in association with Gasul phenomenon. While BAV is common, BPV is extremely rare, especially with defects like VSD. The condition requires detailed imaging and timely surgery to prevent complications such as RV outflow obstruction or heart failure. It underscores the importance of early detection, comprehensive echocardiographic assessment, and high clinical suspicion, as in vivo diagnosis of bilateral bicuspid valves remains exceptionally rare.

### Learning objectives

1. Recognize the clinical significance and rarity of concurrent bicuspid aortic and pulmonary valves, particularly in association with the Gasul phenomenon.
2. Understand the role of detailed echocardiographic and catheterization findings in diagnosing complex congenital heart anomalies.
3. Appreciate the importance of early detection and timely surgical intervention to prevent complications such as RVOT obstruction and heart failure.

## Supplementary Information

Additional file1: Video 01: Apical five chamber view with color flow showing small restrictive perimembranous ventricular septal defect with left to right shunt.

Additional file2: Video 02: Parasternal short axis view at level of aortic valve with color flow showing small restrictive perimembranous ventricular septal defect with left to right shunt along with right ventricular outflow tract muscular stenosis due to infundibular muscle hypertrophy.

## Data Availability

No datasets were generated or analysed during the current study.

## Abbreviations

CHD: Congenital heart disease
BAV: Bicuspid aortic valve
BPV: Bicuspid pulmonary valve
VSD: Ventricular septal defect
ECG: Electrocardiography
2D ECHO: Two dimensional echocardiography
RVOTO: Right ventricular outflow obstruction
NYHA: New York Heart Association
CBC: Complete blood count
RFT: Renal function test
LFT: Liver function test
ASD: Atrial Septal defect
DORV: Double outlet right ventricle
TOF: Tetralogy of Fallot
TGA: Transposition of great arteries
RVH: Right ventricular hypertrophy
